# Acta Academiæ Scientiarum Imperialis Petropolitanæ, pro anno 1777

**Published:** 1784

**Authors:** 


II. *Acta Academiæ Scientiarum Imperialis Petropolitanæ, pro anno* 1777. Pars poſterior. 4to. Petropoli, 1780. 479 pages, with 14 copper plates.

OF the firſt part of this volume, publiſhed in the year 1778, we have already given an account (vol. ii. p. 26). The firſt 84 pages of the part now before us are allotted to the Hiſtory of the Academy. Under this head we meet with the following articles; viz. 1. *Obſervations on the medicinal virtues of ſome indigenous plants, the uſe of which has either been unknown to, or neglected by, the moderns; extracted by Profeſſor* Pallas *from a paper preſented to the Academy by* John Emanuel Gillibert, M. D. *formerly Profeſſor of Anatomy and Botany at Lyons, Director of the Royal Medical Academy at Grodno, &c.* The author of theſe obſervations begins with recommending the leaves of the *Euphorbia Cypariſſias,* dried and powdered, as a ſubſtitute for jalap. Thirty grains of this medicine are ſaid to prove gently purgative to children ſeven years old. In adults the doſe may be increaſed to two ſcruples. The author ſpeaks of this as a remedy of ſingular efficacy in cutaneous diſeaſes. He obſerves that the roots and ſeeds of this

ſpecies of Euphorbia were in uſe amongſt the ancients, and are particularly extolled by Rulandus; but theſe parts of the plant, from their containing a large proportion of reſin, operate with too much violence; an objection, it ſeems, which does not hold good with reſpect to the leaves. As a ſubſtitute for Ipecacoanha, the author recommends *Aſarum* given either in powder (in any quantity from twelve to thirty grains) or in decoction. Twenty grains of the powder mixed with three ounces of manna are ſaid to prove ſtrongly purgative as well as emetic. The root of the *Eupatorium Cannabinum*, formerly recommended by Geſner, has likewiſe been found by our author to be a very mild emetic, and ſpeedy in its operation. He obſerves that the bark of this root excites vomiting, when infuſed either in wine or water; but he gives the preference to it in ſubſtance.

M. Gillibert next ſpeaks of the efficacy of a decoction of Saponaria in viſceral obſtructions. As a ſubſtitute for Cantharides in the way of bliſters, he thinks that ſeveral of the acrid plants might be uſed with ſucceſs. He ſpeaks of an old woman in Poland, who uſed to cure tertian fevers, after the third fit, by applying to the wriſts an epithem compoſed of the freſh bark bark of the *Clematis recta*, which raiſes veſicles without exciting inflammation. He himſelf, he obſerves, has ſeen the external application of the bruiſed leaves of the *Ranunculus acris* procure immediate relief in an obſtinate caſe of *clavus hyſtericus*. To this account Profeſſor Pallas adds, that the peaſants in Ruſſia are accuſtomed to apply as a veſicatory the bruiſed leaves of the *Anemone patens*.

In cutaneous diſeaſes M. Gillibert recommends a decoction of liquorice root (taken as common drink, and likewiſe applied externally in the way of lotion) as a uſeful remedy when aſſiſted by a milk-diet.

2. *Remedy of the Calmucks againſt the Rheumatiſm.*—An interpreter who reſides among the Calmucks, at the expence of the Academy, with a view to acquire information concerning their language, cuſtoms, &c. has communicated this account of a remedy which theſe people have lately begun to make uſe of againſt the rheumatiſm. It conſiſts of a decoction of the *Ephedra Monoſtacha*, a ſmall ſhrub which grows in great abundance in elevated and ſandy ſituations in the ſouthern parts of Ruſſia and Siberia. The patients, who take this medicine, are directed to be well covered in order to favour ſweating, which it excites very profuſely. The complaint, we are told, ſeldom fails to be removed after the firſt or ſecond ſweat. Profeſſor Pallas thinks it probable that the *Ephedra Diſtachya*, which grows in Spain, and the ſouthern provinces of France, poſſeſſes ſimilar virtues.

The Hiſtory of the Academy is followed by the *Acta*. The papers which compoſe this part of the work are arranged under the ſeveral heads of *Mathematica*, *Phyſico-Mathematica*, *Phyſica*, and *Aſtronomica*. The papers contained in the firſt of theſe diviſions are, 1. *Lobeliæ Hybridæ, auctore* J. T. Koelreuter.—In our account of the former part of this volume (vol. ii. p. 33) we noticed the experiments of this ingenious academician on the production of *hybridæ* or mules in the vegetable kingdom. In the preſent paper he gives the reſult of his experiments, with different ſpecies of *Lobelia*.—2. *Diſſertatio de Cranio Rhinocerotis Africani, Cornu Gemino, auctore* Petro Camper (ſee our 3d vol. p. 30).—3. *Additamentum ad precedentem Diſſertationem, auctore* P. S. Pallas.—4. *Obſervatio de Dentibus molaribus foſſilibus ignoti animalis, Canadenſibus analogis, etiam ad Uralenſe jugum repertis.*—5. *Obſervationes circa Myrmecophogam Africanam & Didel-*

*Didelphidis novam speciem orientalem; e Literis celeberr.* Petri Camper *excerptæ & illustratæ.*—6. *Description du Bufle a queue de cheval, precedée d'Observations generales sur les especes sauvages du gros betail.*—7. *Observations sur l'Asne dans son etat sauvage, ou sur le veritable Onagre des Anciens.*—The last four articles are by Professor Pallas.

In reviewing collections like the present we are obliged, by the limits of our work, to confine ourselves, as much as possible, to subjects strictly *medical.* Of articles, like those just now enumerated, we must in general content ourselves with mentioning only the titles.

